# Reproductive health issues in rural Western Kenya

**DOI:** 10.1186/1742-4755-5-1

**Published:** 2008-03-18

**Authors:** Anna M van Eijk, Kim A Lindblade, Frank Odhiambo, Elizabeth Peterson, Evallyne Sikuku, John G Ayisi, Peter Ouma, Daniel H Rosen, Laurence Slutsker

**Affiliations:** 1 Department of Infectious Diseases, Tropical Medicine and AIDS, Academic Medical Centre, University of Amsterdam, The Netherlands; 2 Division of Parasitic Diseases, National Centre for Zoonotic, Vector-Borne and Enteric Diseases, Centers for Disease Control and Prevention, Atlanta GA, USA; 3 Centre for Global Health Research, Kenya Medical Research Institute, P.O. Box 1578, Kisumu, Kenya; 4 Vermont Department of Health, Burlington, VT, USA; 5 Global Aids Program, Centers for Disease Control and Prevention, Zimbabwe

## Abstract

**Background:**

We describe reproductive health issues among pregnant women in a rural area of Kenya with a high coverage of insecticide treated nets (ITNs) and high prevalence of HIV (15%).

**Methods:**

We conducted a community-based cross-sectional survey among rural pregnant women in western Kenya. A medical, obstetric and reproductive history was obtained. Blood was obtained for a malaria smear and haemoglobin level, and stool was examined for geohelminths. Height and weight were measured.

**Results:**

Of 673 participants, 87% were multigravidae and 50% were in their third trimester; 41% had started antenatal clinic visits at the time of interview and 69% reported ITN-use. Malaria parasitemia and anaemia (haemoglobin < 11 g/dl) were detected among 36% and 53% of the women, respectively. Geohelminth infections were detected among 76% of the 390 women who gave a stool sample. Twenty percent of women were underweight, and sixteen percent reported symptoms of herpes zoster or oral thrush in the last two months. Nineteen percent of all women reported using a contraceptive method to delay or prevent pregnancy before the current pregnancy (injection 10%, pill 8%, condom 0.4%). Twenty-three percent of multigravidae conceived their current pregnancy within a year of the previous pregnancy. More than half of the multigravidae (55%) had ever lost a live born child and 21% had lost their last singleton live born child at the time of interview.

**Conclusion:**

In this rural area with a high HIV prevalence, the reported use of condoms before pregnancy was extremely low. Pregnancy health was not optimal with a high prevalence of malaria, geohelminth infections, anaemia and underweight. Chances of losing a child after birth were high. Multiple interventions are needed to improve reproductive health in this area.

## Background

The Millennium Development Goals (MDGs) represent a commitment of countries to address global poverty and ill health, and are intended as a global framework to assess progress in countries and regions [1]. Reproductive health was initially omitted from the Millennium Development Goals; however, it is now recognized as a key issue for all goals [2]. Indirectly, many reproductive health issues are part of the MDGs, e.g., reduction of child and maternal mortality and improved maternal health (goals number 4 and 5, respectively). In addition, the promotion of gender equality and empowerment of women (goal 3) has a vital role. Adequate reproductive health services and family planning are essential. Combating HIV/AIDS, malaria, and other diseases (Goal 6), is particularly relevant in the setting of sub-Saharan Africa.

Maternal and infant health have greatly improved in developed countries where infant mortality, adverse neonatal outcomes and maternal mortality have been substantially reduced [3,4]. This improvement has been accompanied by an increase in the use of contraceptives and a decrease in fertility rates in these countries [5]. However, many developing countries have not seen such a progress. In Kenya, the total fertility rate is approximately 4.9 and has not decreased since 1998; approximately one in 5 pregnancies is unwanted [6]. Maternal care and care of the newborn are often less than optimal; the proportion of home deliveries is high and has not changed since 1998; infant and under-5 child mortality are still increasing [6]. Diseases such as malaria and HIV contribute to adverse pregnancy outcomes, despite increasing treatment and/or prevention options.

Multiple factors are known to be important for reproductive health, including socio-economic status, social values, and accessibility and quality of health care. During a survey in December 2002 among women who had recently delivered in rural western Kenya, we examined the use of antenatal services and delivery care and reported that despite the high antenatal clinic attendance (90% visited at least once), only seventeen percent of women delivered with a skilled attendant whereas 18% delivered completely on their own [7]. A subsequent survey in July 2003 among pregnant women in the same area allowed us to describe pre-pregnancy contraceptive use, reproductive history, pregnancy health and nutritional status, thus further complementing our knowledge on reproductive health in rural western Kenya.

## Methods

### Study site

This study was conducted in Wagai and Yala (Gem) Divisions in Nyanza province in July 2003; the study area has been described previously [8]. Gem has a population of 75,000 people residing in 142 villages. This area previously suffered from intense, perennial malaria transmission. However, provision of free insecticide-treated bednets (ITNs) as part of a community-randomized controlled trial from 1997–2002 reduced transmission by 90% along with significant reductions in infant mortality and morbidity, as well as malaria in pregnancy [9-11]. The community continued to receive free ITNs and retreatment after the conclusion of the study [12]. Since May 2002, this area has been under continuous demographic surveillance [13]. All births, deaths, pregnancies, and in- and out migrations are updated every four months; socio-economic information and educational level are regularly recorded. The age-adjusted prevalence rates of HIV in men and women 13–34 years old in this area are 11% and 21%, respectively (P. Amornkul, personal communication); the Demographic and Health survey in 2003 reported an HIV prevalence in Nyanza province of 15% (18% among women and 12% among men) [6]. The total fertility rate in this area calculated for 2002 was 5.3 live births [13].

### Study participants

For this community-based survey, a sample of a list of all pregnant women was generated from the demographic surveillance system (DSS). Based on previous estimates, we anticipated that at least 600 women in Gem would be pregnant at the time of the survey. We estimated that we would need a sample of approximately 403 women, allowing for 20% non-response and based on stratification by gravidity (women with ≤ 4 pregnancies vs. > 4 pregnancies) to calculate the true proportion of those anaemic or parasitemic within 5 percentage points of the true proportion. Pregnant women were invited to attend the survey by community health workers who visited their area a couple of days before the survey. They were provided with a stool cup and asked to bring a fresh stool sample at the day of the survey. In the first week of the survey it was noted that the time lapse between collection of pregnancy data and the timing of the survey resulted in the majority of women having already delivered. Therefore, from the second week onwards, community health workers in each village invited all pregnant women in their village who were known to them to participate in the survey. At the end of the survey, we returned to the villages which were visited in the first week to allow pregnant women who were not known to the DSS in these areas to be enrolled.

### Procedures

After informed consent, trained study staff interviewed participants to obtain socio-demographic information, and a reproductive and medical history. We asked for the use of a contraceptive method to prevent or delay pregnancy, but did not ask if the pregnancy was the result of failed contraception. Weight was recorded on a weighing scale and height was measured to the nearest centimetre on a locally made height instrument. The mid upper arm circumference (MUAC) was measured using a pre-printed arm tape to the nearest 0.1 cm. Gestational age was estimated by abdominal palpation by a clinical officer. In addition, we recorded the first day of the last menstrual period if known. Blood was obtained by finger stick for a haemoglobin level and a thick and thin blood smear. The clinical officer treated pregnant women according to history and physical findings; a limited drug supply was available on site. All women with anaemia (haemoglobin < 11 g/dl) were treated with iron tablets, (200 mg three times a day for one month) and folic acid tablets (5 mg once a day for one month). If pregnant women were eligible but had not yet received intermittent preventive treatment with sulfadoxine-pyrimethamine for malaria, they were provided this by the clinical officer as per the national guidelines. Pregnant women identified as acutely ill and needing admission were referred and transported to a hospital in the area.

Blood smears were transported to a central laboratory where they were stained the next day using Giemsa. All blood smears were examined under oil immersion: parasite count and gametocyte counts were determined against 300 and 500 white blood cells respectively. A 10% sample of all smears was read by a different examiner to check the quality of the smear reading. Malaria parasite densities were estimated by assuming a count of 8000 WBC per microliter of blood. A thick blood smear was considered negative if 100 microscopic fields revealed no parasites. Haemoglobin was measured using a Hemocue^® ^haemoglobin detection system (HemoCue AB, Angelholm, Sweden) at the field site. The stool sample was examined for ova and cysts of parasites, using Kato Katz and formal-ether stool concentration method.

### Definitions and data analysis

Anaemia was defined as a haemoglobin < 11 g/dl and severe anaemia as a haemoglobin < 8 g/dl. Documented fever was defined as an axillary temperature ≥ 37.5°C. Malaria parasitemia was defined as the presence of any asexual blood stage parasite of any species in a thick smear. The body mass index was calculated as weight (kg)/height (meters) squared; a low body mass or underweight was defined as a body mass index less than the average 5^th ^percentile of the body mass index by trimester of a Swiss reference population (18.8 kg/m^2 ^for the first trimester, 20.2 kg/m^2 ^for the second and 22.3 kg/m^2 ^for the third trimester) [14]. A low mid upper arm circumference (MUAC) or wasting was defined as a mid upper arm circumference less than 22 cm [15]. We used a history of herpes zoster or oral thrush in the last two months as an indication of HIV-infection [16]. Socio-economic information was obtained from the demographic surveillance system (DSS). Information on household assets was used to derive a wealth index using principal component analysis [17]. All households were categorized by quintile: a medium/low socio-economic status (SES) was defined as an SES in the bottom 3 quintiles of the wealth index. A woman less than 20 years of age was considered a young woman. Modern contraceptives included the use of the contraceptive pill, an injection, IUD, or condom as contraceptive method. We defined ITN use as the use of an insecticide treated net for 5 or more nights per week. Differences in proportions were analysed using the chi-square test or Fisher's exact test when appropriate. Differences in means were compared by Student's *t*-test or the Wilcoxon rank test where appropriate. We used logistic regression to assess the association between the use of a modern contraceptive method and the following characteristics: age, gravidity, SES, marital status and education level. Only factors with a *P*-value < 0.05 were maintained in the multivariate model. SAS (SAS system for Windows version 9, SAS, Cary, NC) was used for all analyses. All tests were two-sided; *P *< 0.05 was considered significant.

The protocol for the community-based survey was reviewed and approved by the institutional review boards of the Kenya Medical Research Institute (Nairobi, Kenya) and the Centers for Disease Control and Prevention (Atlanta, GA, USA). All women who participated gave written informed consent after reading through the consent form with the interviewer; participants who could not write indicated their consent by a fingerprint in the presence of a witness.

## Results

### Characteristics

A total of 673 pregnant women participated; 87% were multigravidae (Table 1). Of the 582 women who reported being married, 128 (22%) reported their husband was not living in the same household. Among married women, 104 (18%) reported being co-wives (wives who were married according to local traditional customs to the same husband). Of the participating women, 84% could be matched with the local DSS for educational level, and 77% for socio-economic status. Women who could not be matched were more likely to be younger and to have a lower gravidity number compared to women who were matched (data not shown). Only 28 (5%) of the 563 women whose educational status was known did not attend any school.

**Table 1:** Characteristics of participating pregnant women, Gem, western Kenya, 2003

**Demographic and socio-economic information**	**Total, n (%) (N = 673)**
Mean age (SD), years	26.2 (6.7)
Age	
< 20 years	123 (18.3)
20–29 years	361 (53.6)
≥ 30 years	189 (28.1)
Median gravidity (range)	4 (1–15)
Gravidity	
Primigravidae	88 (13.1)
Gravidae 2–4	333 (49.5)
Gravidae ≥ 5	252 (37.4)
Trimester of pregnancy*	
First	42 (6.3)
Second	292 (43.6)
Third	336 (50.2)
Married	582 (86.5)
Education < 8 years*	332 (59.0)
Low/medium SES*	352 (68.1)
< 3 meals per day	244 (36.3)

Abbreviations: SD, standard deviation; SES, socio-economic status;

* Trimester not known for 3 women (0.4%); education level not known for 110 women (16.3%); SES category not known for 156 women (23.2%).

### Use of contraceptives before pregnancy

Hundred and twenty seven women (19%) reported having used a method to delay or prevent pregnancy before their current pregnancy; an injection was the most common mentioned method (66 women or 10% of all participants), followed by oral contraceptives (53 women or 8%), traditional methods (10 women), abstinence (8 women), IUD (4 women), condoms (3 women), and other methods (1 woman). Sixteen women reported the use of more than one type of contraceptive. Only 3 primigravidae and 7 women younger than 20 years reported the use of a contraceptive method (*P *< 0.01).

Modern contraceptives were reported by 117 women (17%). Their use was independently associated with age, gravidity, and education level (Table 2).

**Table 2:** Factors associated with the use of modern contraceptive methods among women, Gem, western Kenya, 2003*

	**Use, n (%)**	**OR (95% CI)**	**AOR (95% CI)†**
Gravidity			
Primigravidae	2 (2.3)	**0.07 **(0.02–0.28)	**0.05 **(0.01–0.34)
Gravidae 2–4	50 (15.0)	**0.51 **(0.34–0.77)	**0.47 **(0.29–0.75)
Gravidae ≥ 5	65 (25.8)	Reference	Reference
Age			
< 20 years	6 (4.9)	**0.16 **(0.06–0.38)	
20–29 years	64 (17.7)	**0.65 **(0.43–0.99)	
≥ 30 years	47 (24.9)	Reference	
Education level‡			
< 8 years	44 (13.3)	**0.58 **(0.37–0.91)	**0.51 **(0.32–0.81)
≥ 8 years	48 (20.8)	Reference	Reference
Marital status			
Married	109 (18.7)	**2.39 **(1.12–5.09)	
Not married	8 (8.8)	Reference	
SES‡			
Lower 3 quintiles	54 (15.3)	0.75 (0.47–1.22)	
Higher 2 quintiles	32 (19.4)	Reference	

Abbreviations: OR: odds ratio, AOR: adjusted odds ratio, CI: confidence interval, SES: socioeconomic status. Significant odds ratios are printed in bold.

*Modern contraceptive methods included oral contraceptives, the injection for contraceptive use, the intrauterine device and condoms. These were reported by 117 women (17.4% of the study population).

† Because of co-linearity, age not included in this model. In a model with age instead of gravidity, the adjusted odds ratios are: 0.16, 95% CI 0.06–0.43, for age < 20 years, and 0.55, 0.34–0.89, for 20–29 years (age ≥ 30 years as reference group).

‡ Education level: 110 missing (16.3%); SES: 156 missing (23%).

### Reproductive and obstetric history

Of 585 multigravidae, 174 (30%) reported they were breastfeeding when they became pregnant. More than half (55%) had lost a live born child (range of number of children lost: 1–7) (Table 3). The experiences ever in life of a miscarriage, stillbirth, a too small child, a premature child, or loss of a life born child were not associated with marital status, level of education or SES. Of the 558 multigravidae with known birth interval, 127 (23%) conceived the current pregnancy within a year of the previous pregnancy. A short birth interval (< 1 year) was associated with a young age (43% vs. 21%, *P *< 0.01) and outcome of the previous pregnancy; 14% among women whose previous birth was a live born child who was still alive, 43% among women whose live born child died, and 81% among women whose previous pregnancy resulted in a miscarriage or stillbirth (*P *< 0.01). Socio-economic or marital status and level of education were not related to a short birth interval (data not shown).

**Table 3:** Outcome of previous pregnancies and birth interval among multigravidae, Gem, western Kenya, 2003

	**Outcome of last singleton pregnancy (%) (N = 573)**	**Outcome ever in life (%) (N = 585)**
Miscarriage	14 (2.4)	67 (11.5)
Stillbirth	9 (1.6)	53 (9.1)
Child death of live born child	120 (20.9)	322 (55.0)
Caesarean section	NA	11 (1.9)
Too small baby	17 (3.1)*	82 (14.0)
Baby born too early	21 (3.8)*	111 (19.0)
		
Birth interval †	**All multigravidae **‡	
< 6 months	54 (9.7)	
6–11 months	73 (13.1)	
12–23 months	198 (35.5)	
≥ 24 months	233 (41.8)	

NA: not available.

* Size of newborn and estimated gestational age known for 550 newborns

† Birth interval: time between last menstrual period and previous delivery date

‡ Birth interval available for 558 multigravidae (95.4%), independent of number of children in previous delivery

### Health issues during pregnancy

**Figure 1:**
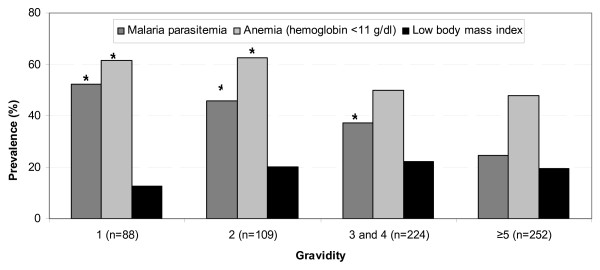
**Prevalence of malaria, anaemia and underweight among pregnant women in Gem, western Kenya, 2003**. *P < 0.05 compared to gravidae ≥ 5. For a definition of low body mass index see the Methods section under "Data analysis".

Smoking and the use of alcohol were not common in this population, reported by 2 and 23 women, respectively. Twenty percent of the women had a low BMI, and 2.5% had a low MUAC; the prevalence of a low MUAC did not differ by gravidity but a low BMI was less common among primigravidae (12% vs. 21% among multigravidae, *P *= 0.08) (Figure 1). Over one third of the women had malaria parasitemia (36%) and over half of the participants were anaemic (53%, Table 4); the prevalence of malaria and anaemia was significantly higher among women in their first and second pregnancy compared to gravidae ≥ 5 (malaria: 52% and 46% among primigravidae and secondigravidae respectively, and anaemia: 61% and 62%, Figure 1). Malaria was less prevalent among women who used an ITN (31% vs. 47%, *P *< 0.01). Helminthic infections were extremely common (76%) among the 390 women who gave a stool sample, with *Ascaris lumbricoides *as the most frequently detected species (52%), followed by hookworm (39%) and *Trichuris trichiura *(29%).

**Table 4:** Health among pregnant women and care used, overall and by age group, Gem, western Kenya, 2003

	**Total**	**Age group (years)**		
	**N (%)**	**< 20**	**20–29**	**≥ 30**
Malaria*	241 (35.9)	63 (51.2)	134 (37.1)	44 (23.4) †
Anaemia (Hb < 11 g/dl)	355 (52.8)	68 (55.3)	192 (53.2)	95 (50.3)
Severe anaemia (Hb < 8 g/dl)	59 (8.8)	7 (5.7)	33 (9.1)	19 (10.1)
Low BMI*	131 (19.6)	11 (8.9)	85 (23.7)	35 (18.5) †
MUAC < 22 cm	17 (2.5)	2 (1.6)	10 (2.8)	5 (2.7)
Geohelminth infection*	297 (76.2)	54 (76.1)	146 (75.3)	97 (77.6)
Oral thrush or herpes zoster in the last 2 months	108 (16.1)	17 (13.8)	70 (19.4)	21 (11.1) †
Complaint in previous 2 weeks of:				
Fever	421 (62.6)	68 (55.3)	231 (64.0)	122 (64.6)
Cough	385 (57.2)	63 (51.2)	217 (60.1)	105 (55.6)
Diarrhoea	124 (18.4)	25 (20.3)	61 (16.9)	38 (20.1)

				

**Care during pregnancy**				
ANC started	277 (41.2)	58 (47.2)	162 (44.9)	57 (30.2) †
Bed net in house	509 (75.6)	77 (62.6)	261 (72.3)	171 (90.5) †
Used ITN	465 (69.1)	64 (52.0)	239 (66.2)	162 (85.7) †

Abbreviations: Hb, haemoglobin; BMI, body mass index; MUAC, mid-upper arm circumference; ANC, antenatal clinic; ITN: insecticide treated net.

* Blood smear not available for 1 woman (0.1%); BMI missing for 3 women (0.4%); stool result not available for 283 women (42.1%)

† Chi-square test: *P *< 0.05

The prevalence of oral thrush or herpes zoster in the last two months was 16%. Twenty five women (4%) reported to have been admitted to the hospital during the present pregnancy; malaria was the most common reason given for admission (9 women).

### Bednet use and antenatal care during pregnancy

Of all women, 509 (76%) reported having a bed net in the house; 97% of these nets were treated with insecticide, and 93% of women reported sleeping under the net more than 5 times per week. In the total study population, 69% of women used an ITN regularly. The use of ITN was higher by age and gravidity (Table 4, data not shown for gravidity), but otherwise not related to SES, level of education or marital status.

Only 41% of women had started antenatal clinic visits; this proportion was higher in the third (61%), compared to the second (24%) and first trimester (7%, *P *< 0.01); and was also higher among primigravidae (61%) than multigravidae (38%, *P *< 0.01). Marital status and level of education were not associated with start of ANC visits, however, women of lower/medium SES were less likely to have started (34.4%) compared to women with higher SES (48%, *P *< 0.01). The variables trimester, gravidity and SES remained significantly associated with antenatal clinic visit in multivariate analysis (data not shown).

Among the 277 women who had visited an ANC, 125 (45%) had started taking iron supplementation, 120 (43%) had started taking folic acid supplementation, and 246 (89%) had received a tetanus injection. Of the 263 women who were in 2^nd ^or 3^rd ^trimester and had visited ANC, 51 had received sulfadoxine-pyrimethamine (19%) for malaria; for the total study population this proportion was 8%.

## Discussion

Among pregnant women in this rural area with a high prevalence of malaria and HIV, approximately 1 in 5 women had used a contraceptive prevention method before their most recent pregnancy. Malaria, anaemia, helminthic infections and complaints of cough and fever were common. One in 5 women was underweight, although the proportion of wasting was low (2.5%). The use of bed nets was high due to the previous ITN trial in the area. However, less than half of all women had started antenatal clinic visits even though the majority were past their first trimester. Only 8% of women beyond their first trimester had received a dose of sulfadoxine-pyrimethamine.

Although the reported use of contraceptives before the current pregnancy was not common (18.9%), we did not directly ask if the current pregnancy was a result of failed contraception. A similar rate of contraceptive use among married women was reported in the 2003 demographic health survey in Kenya for Nyanza Province (21%); however, our results are not directly comparable to those because we only interviewed pregnant women [6]. The 2003 demographic health survey reports that 76% of women in Nyanza Province have had exposure to messages about condoms, mainly through the radio; however, they report a similar low use of condoms (0.3%) for this region [6]. Given the very high prevalence of HIV in this area, the very low prevalence of condom use among sexually active women is of concern. Assuming that all women who reported oral thrush or herpes zoster in the previous months were HIV-infected, the proportion of HIV-infected pregnant women in the survey could be at least 16%. This is likely to be an underestimate; as mentioned before, a survey in a neighbouring area revealed age-adjusted prevalence rates of HIV of 21% among women aged 13–34 years old (P. Amornkul, personal communication). In this area with a high HIV-prevalence, HIV prevention and treatment programs are available and expanding despite the challenge of stigma, costs and logistics [18,19].

Multigravide women had a disturbing reproductive history with a high reported rate of stillbirth, growth retardation, prematurity, and previous loss of a live born child. Over 20% reported they had already lost their last live born child by the time of the survey. This is indicative of the very high infant and under-five child mortality rates in the area, which has been estimated at 125 and 227 per 1,000 live births for the DSS area (Gem and Asembo) [13]. Factors during pregnancy and delivery, such as malaria, anaemia, and HIV are likely to contribute to infant mortality through their contribution to low birth weight, prematurity and stillbirth. However, post natal factors such as malaria, anaemia, diarrhoea, respiratory infections, and sepsis are also important for infant mortality, as evidenced by verbal autopsy data from the same area [13]. Thus, multiple simultaneous interventions are needed to improve the reproductive outcomes among these women [20,21].

From the perspective of pregnancy health, multiple interventions are available that can help improve pregnancy outcome and infant survival. Malaria, anaemia and helminthic infections can be addressed by intermittent preventive treatment with sulfadoxine-pyrimethamine, iron and folic acid supplementation, and antihelminthic treatment in antenatal clinics. Unfortunately, women in this area delay their first antenatal clinic visit, missing an early opportunity to benefit from these treatments. Moreover, the proportion of women who had started antenatal visits and had received sulfadoxine-pyrimethamine, iron, and folic acid was not optimal, suggesting opportunities for improvement.

The benefits of the use of ITNs in pregnancy has been recently reviewed [22]. Although the use of ITNs in this population was high and associated with a reduction in parasitemia, the prevalence of parasitemia was still considerable. Additional use of intermittent preventive treatment with sulfadoxine-pyrimethamine from the antenatal clinics may be necessary to optimize malaria control in pregnancy in this area. The effect of the combination of sulfadoxine-pyrimethamine with ITNs for malaria in pregnancy has not yet been well documented [23].

Only 2 % of the women ever had a caesarean section, and 9% ever had a stillbirth. The number of stillbirths has been correlated with maternal mortality in one ecological study, with about 2 to 5 stillbirths for each maternal death in a developing country [24]. The same study showed that when caesarean section rates increased from 0 to 10%, both maternal mortality and stillbirths sharply decreased [24]. Stillbirth and caesarean section rates highlight areas where additional efforts may make pregnancy safer.

Our study had limitations. Because of sampling problems, we invited every known pregnant woman in the area to the survey. We cannot exclude that this may have biased our results to women who were not afraid to declare themselves pregnant and were known to the community interviewer. However, the health benefits observed by pregnant women during the survey may have resulted in women declaring their pregnancy earlier than usual, thus surpassing expected numbers from the DSS. In particular young women could not be matched with data available from the DSS, supporting observations that newly married wives may have recently moved in from outside the area, and were not yet enrolled in the DSS. We did not obtain detailed information on other health factors which have been shown to be important for pregnancy outcome, such as syphilis or high blood pressure [21,25]. For the assessment of underweight we used the BMI norms of a Swiss population as reference group; however, the same study also evaluated effect of race on BMI in pregnancy and concluded that BMI norms for Caucasians can be used for people of African origin [14]. The MUAC is a common tool to evaluate nutritional status among African women [15,26].

It is obvious there are many challenges in this area to achieve the MDGs of reduction of child mortality, improvement of maternal health and combating HIV/AIDS, malaria and other diseases. Programs are underway to decrease HIV-transmission, and improve the care for HIV-infected persons. In the antenatal clinics, programs have been implemented to improve maternal health and decrease mother-to-child transmission of HIV. ITNs are promoted and used. Family planning programs are present in the area. It will be important to continue evaluating if the existing programs are optimally functioning, and if the target group is optimally using them; in addition, if programs are not used, it is important to evaluate what barriers exist, and how programs can be changed to achieve their goals.

## Conclusion

In this rural area with a high HIV prevalence, the reported use of condoms before pregnancy was extremely low. Pregnancy health was not optimal, with a high prevalence of infections and considerable underweight. Among multigravidae, the reports of adverse pregnancy outcomes were high; the reproductive histories of these women showed that even after a successful delivery of a live born child, the mortality risk for this child in this area remains high. Multiple simultaneous interventions are needed to address these challenges.

## Competing interests

The author(s) declare that they have no competing interests.

## Authors' contributions

AMvE was involved in the design and conduct of the study, in analysis and drafting the paper. KAL and LS were involved in conception and design, interpretation of the data and review of the manuscript. EP, FO, ES, and JGA were involved in data collection, and revision of the manuscript. PO was involved in analysis and drafting the paper. DHR was involved in the statistical analysis, interpretation of the data and review of the manuscript. Each author has given final approval of the version to be published.

## References

[B1] Haines A, Cassels A (2004). Can the millennium development goals be attained?. BMJ.

[B2] Glasier A, Gulmezoglu AM, Schmid GP, Moreno CG, Van Look PF (2006). Sexual and reproductive health: a matter of life and death. Lancet.

[B3] Ronsmans C, Graham WJ (2006). Maternal mortality: who, when, where, and why. Lancet.

[B4] Lawn JE, Cousens S, Zupan J (2005). 4 million neonatal deaths: when? Where? Why?. Lancet.

[B5] Lutz W, Qiang R (2002). Determinants of human population growth. Philos Trans R Soc Lond B Biol Sci.

[B6] Central Bureau of Statistics Kenya, ORC Macro (2004). Kenya Demographic and Health Survey 2003.

[B7] van Eijk AM, Bles HM, Odhiambo F, Ayisi JG, Blokland IE, Rosen DH, Adazu K, Slutsker L, Lindblade KA (2006). Use of antenatal services and delivery care among women in rural western Kenya: a community based survey. Reprod Health.

[B8] Phillips-Howard PA, Nahlen BL, Alaii JA, ter Kuile FO, Gimnig JE, Terlouw DJ, Kachur SP, Hightower AW, Lal AA, Schoute E, Oloo AJ, Hawley WA (2003). The efficacy of permethrin-treated bed nets on child mortality and morbidity in western Kenya I. Development of infrastructure and description of study site. Am J Trop Med Hyg.

[B9] Gimnig JE, Vulule JM, Lo TQ, Kamau L, Kolczak MS, Phillips-Howard PA, Mathenge EM, ter Kuile FO, Nahlen BL, Hightower AW, Hawley WA (2003). Impact of permethrin-treated bed nets on entomologic indices in an area of intense year-round malaria transmission. Am J Trop Med Hyg.

[B10] Phillips-Howard PA, Nahlen BL, Kolczak MS, Hightower AW, ter Kuile FO, Alaii JA, Gimnig JE, Arudo J, Vulule JM, Odhacha A, Kachur SP, Schoute E, Rosen DH, Sexton JD, Oloo AJ, Hawley WA (2003). Efficacy of permethrin-treated bed nets in the prevention of mortality in young children in an area of high perennial malaria transmission in western Kenya. Am J Trop Med Hyg.

[B11] ter Kuile FO, Terlouw DJ, Phillips-Howard PA, Hawley WA, Friedman JF, Kariuki SK, Shi YP, Kolczak MS, Lal AA, Vulule JM, Nahlen BL (2003). Reduction of malaria during pregnancy by permethrin-treated bed nets in an area of intense perennial malaria transmission in western Kenya. Am J Trop Med Hyg.

[B12] Lindblade KA, Eisele TP, Gimnig JE, Alaii JA, Odhiambo F, ter Kuile FO, Hawley WA, Wannemuehler KA, Phillips-Howard PA, Rosen DH, Nahlen BL, Terlouw DJ, Adazu K, Vulule JM, Slutsker L (2004). Sustainability of reductions in malaria transmission and infant mortality in western Kenya with use of insecticide-treated bednets: 4 to 6 years of follow-up. JAMA.

[B13] Adazu K, Lindblade KA, Rosen DH, Odhiambo F, Ofware P, Kwach J, AM VANE, Decock KM, Amornkul P, Karanja D, Vulule JM, Slutsker L (2005). Health and Demographic Surveillance in Rural Western Kenya: a Platform for Evaluating Interventions to Reduce Morbidity and Mortality from Infectious Diseases. Am J Trop Med Hyg.

[B14] Ochsenbein-Kolble N, Roos M, Gasser T, Zimmermann R (2007). Cross-sectional study of weight gain and increase in BMI throughout pregnancy. Eur J Obstet Gynecol Reprod Biol.

[B15] James WP, Mascie-Taylor GC, Norgan NG, Bistrian BR, Shetty PS, Ferro-Luzzi A (1994). The value of arm circumference measurements in assessing chronic energy deficiency in Third World adults. Eur J Clin Nutr.

[B16] Morgan D, Mahe C, Mayanja B, Whitworth JA (2002). Progression to symptomatic disease in people infected with HIV-1 in rural Uganda: prospective cohort study. BMJ.

[B17] Filmer D, Pritchett LH (2001). Estimating wealth effects without expenditure data--or tears: an application to educational enrollments in states of India. Demography.

[B18] Volmink J, Siegfried NL, van der Merwe L, Brocklehurst P (2007). Antiretrovirals for reducing the risk of mother-to-child transmission of HIV infection. Cochrane Database Syst Rev.

[B19] Reithinger R, Megazzini K, Durako SJ, Harris DR, Vermund SH (2007). Monitoring and evaluation of programmes to prevent mother to child transmission of HIV in Africa. BMJ.

[B20] Jones G, Steketee RW, Black RE, Bhutta ZA, Morris SS (2003). How many child deaths can we prevent this year?. Lancet.

[B21] Adam T, Lim SS, Mehta S, Bhutta ZA, Fogstad H, Mathai M, Zupan J, Darmstadt GL (2005). Cost effectiveness analysis of strategies for maternal and neonatal health in developing countries. BMJ.

[B22] Gamble C, Ekwaru JP, ter Kuile FO (2006). Insecticide-treated nets for preventing malaria in pregnancy. Cochrane Database Syst Rev.

[B23] ter Kuile FO, van Eijk AM, Filler SJ (2007). Effect of sulfadoxine-pyrimethamine resistance on the efficacy of intermittent preventive therapy for malaria control during pregnancy: a systematic review. JAMA.

[B24] McClure EM, Goldenberg RL, Bann CM (2007). Maternal mortality, stillbirth and measures of obstetric care in developing and developed countries. Int J Gynaecol Obstet.

[B25] Deperthes BD, Meheus A, O'Reilly K, Broutet N (2004). Maternal and congenital syphilis programmes: case studies in Bolivia, Kenya and South Africa. Bull World Health Organ.

[B26] Villamor E, Msamanga G, Urassa W, Petraro P, Spiegelman D, Hunter DJ, Fawzi WW (2006). Trends in obesity, underweight, and wasting among women attending prenatal clinics in urban Tanzania, 1995-2004. Am J Clin Nutr.

